# A Study on Flipped Learning Concerning Learning Motivation and Learning Attitude in Language Learning

**DOI:** 10.3389/fpsyg.2021.753463

**Published:** 2021-09-22

**Authors:** Chi-Pu Chou, Kuo-Wei Chen, Chia-Jen Hung

**Affiliations:** ^1^Department of Business Administration, Chung Yuan Christian University, Taoyuan, Taiwan; ^2^Department of Hospitality Management, Ming Chuan University, Taoyuan, Taiwan; ^3^Department of Food and Beverage Management, Taipei University of Marine Technology, Taipei, Taiwan

**Keywords:** flipped learning, language learning, learning motivation, learning attitude, cognition component, affection component, behavioral tendency

## Abstract

From the popularity of flipped teaching in United States primary and high schools, it is thought that students have more learning control to adjust to the learning progress and are assisted in problem solving and learning guidance during class period. It is believed that flipped teaching could prompt underachieving learners’ active learning and thereby enhance learning effectiveness. A total of 386 high school students in Chungli, Taiwan, were part of an experimental study and the research results are summarized below: (1) Students who participated in the flipped teaching models demonstrated better comprehension levels with the teaching content due to this change in learning style and attitude, which in turn, enhanced learning effectiveness. (2) To eliminate poor language performance of underachieving students, it is necessary to lay solid foundations to gradually enhance language learning effectiveness regarding this particular group of students. Films suitable for students’ individual ability could be combined with new language learned in the unit to genuinely assist underachieving learners’ language learning effectiveness. (3) For students who care about their performance, a “system of play” style grouping should be determined in order to enable the tracking of group performance and term performance. According to the results, further developments regarding active learning ability, boosts in learning interests, enhanced learning effectiveness, and the prompting of creativity resulting in a shift from passive learner to active learner have been proposed.

## Introduction

Due to serious global competition, countries throughout the world are positively changing their domestic education models to enhance national competitiveness. In education reform policies, the use of technology or e-learning has become prevalent. In this way, domestic educational initiatives are provided equally regardless of socioeconomic status and region; the principle of teaching students in accordance with aptitude expects to provide learning styles suitable for students with distinct intelligence. Traditional teaching sites are limited to instructors and teaching hours which can make it difficult to achieve the goals of teaching without partiality and teaching students in accordance with aptitude. E-learning is not restricted to time and space and could precede individualized instruction. In addition to thinking of teachers’ techniques, different levels of ability to teach students are also considered in various countries. According to current education goals in the world, it is of primary importance to cultivate students’ reading ability, mathematical ability, and scientific literacy, encourage cooperation with others, and develop problem-solving abilities.

In order to help slow learners or students not able to keep up due to missing classes, teachers introduced a new teaching approach in their lessons, aiming to have more adaptive classes; students could adjust to the learning progress according to their personal learning pace. Teachers first record the full lessons for students learning at home; the major course content is covered in the teaching films. Task-based learning activity or learning content questioning and discussion are preceded in the classroom. Teachers do not lecture like in formally structured lessons of the past but instead focus mainly on teacher–student interaction. In this manner of teaching, lots of time is allocated for group discussion, homework guidance, and critical thinking in class; besides adaptive instruction could be preceded for guiding slow learners. Such a teaching method is known as flipped teaching.

Chukwuemeka et al. (2021) stated that while suffering from COVID-19, many countries decided to give distance learning a try. Ways of getting an education have had to modify due to the epidemic crisis, but it also sheds some new light on education. It helps teachers to think out of the box and provide a more diversified way of teaching. The range of e-learning has crept up and shown its advantage during COVID-19. The pandemic has brought not only crisis to humans, but also chances (Nerantzi, 2020). We live in a modern society with computers, the internet, and artificial intelligence constantly developing. In the process of producing e-learning teaching materials, flipped learning shows a different technical mean and education method from the traditional ones. It can be seen that students sing the praises of the contents of e-learning and the teachers’ wisdom and responsibilities (Drozdikova-Zaripova and Sabirova, 2020). The concerned departments of education gather teachers with e-learning experience to record video courses of online teaching introduction and online flipped teaching implementation guidelines for students that are in home quarantine during the epidemic prevention period. They also collect e-learning resources, platforms, and tools for teachers and students, so that they can arrange the course or self-study (Umutlu and Akpinar, 2020).

Flipped learning in language learning induces the learning interests and more effectively enhances the learning effectiveness and inspires creativity in changing passive learners into active learners.

## Literature Review and Hypotheses

Chang and Hwang (2018) regarded the main value of flipped classroom as changing class hours into the form of a workshop and having students test their application knowledge through inquiry and mutual discussion. Hence, teachers become coaches or consultants encouraging students to participate in group discussion or individual inquiry. Therefore, flipped learning could better enhance students’ learning motivation and attitude than traditional teaching. Bakla (2018) explained it as promoting students’ active learning, including textbook text reading, taking preview notes, establishing Google teacher–student collaboration platforms, asking students to view teaching films in advance, establishing Facebook communities or groups for teacher–student discussion, and establishing an online evaluation system for students’ answering or filling in self-learning checklists. The results revealed the promotion of students’ learning effectiveness and learning motivation. Lin et al. (2018) combined a flipped classroom with mobile learning for mathematics teaching in elementary schools. The results showed that flipped learning, in comparison with traditional teaching, enhanced students’ learning interests and motivation as well as promoted students’ learning effectiveness; meanwhile, teachers and students presented a positive evaluation concerning the advantages of flipped learning. The following hypothesis is therefore proposed in this study.

**H1:** Flipped learning would affect learning motivation.

Zhang (2019) proposed a flipped classroom and requested that students read textbooks, handouts, or PPT before the class to preview relevant data in the lesson; a lot of class time was then saved for students asking questions and analyzing cases. Such a learning style received students’ positive support to prove that students preferred the learning style of the flipped classroom, compared to traditional learning styles. Aiming at teachers with the practice of flipped learning, Alexander (2018) considered that flipping enhanced job satisfaction and students made progress in learning performance. Moreover, teachers noted the obvious improvement of students’ learning attitude; some pleased teachers revealed that they would continuously apply the flipped learning model. Karabulut et al. (2018) indicated that the effectiveness of flipped learning was not simply on academic performance but could also enhance cooperation and thinking among students; meanwhile, it could change students’ attitude toward learning and teacher–student interaction. Many teachers therefore would like to apply these new teaching methods. In this case, the following hypothesis is proposed and testified in view of the present study.

**H2:** Flipped learning would affect learning attitude.

Chen et al. (2019) proposed the positive correlation between learning motivation and learning attitude; when learning motivation was not satisfied, good learning attitude would not be forthcoming. Awidi and Paynter (2019) regarded positive correlations between learners’ learning motivation and learning attitude, i.e., the stronger the learning motivation, the higher the learning attitude of learners. Green (2019) found that the higher the learning motivation, the higher the learning attitude; learning motivation presented predictability on learning attitude, which was not only the indicator of learning attitude and learning outcome, but also the main indicator to induce learning motivation and curriculum development. Accordingly, the following hypotheses are proposed and testified in view of the present study.

**H3:** Learning motivation presents significant and positive effects on the cognition component of learning attitude.

**H4:** Learning motivation presents significant and positive effects on the affection component of learning attitude.

**H5:** Learning motivation presents significant and positive effects on behavioral tendency in learning attitude.

## Research Method

### Measurement of Research Variables

#### Learning Motivation

Referring to Chang et al. (2019), learning motivation is divided into intrinsic motivation and extrinsic motivation in this study. In the Likert 7-point scale, 1 refers to extremely disagree and 7 refers to extremely agree. The overall reliability coefficients of intrinsic motivation and extrinsic motivation appear to be 0.86 and 0.85, respectively.

#### Learning Attitude

Referring to Cheng and Tsai (2019), learning attitude contains a cognition component, affection component, and behavioral tendency with a Likert 7-points scale, 1 refers to the response of extremely disagree and 7 refers to the response of extremely agree. The overall reliability coefficients of learning attitude show 0.82 for the cognition component, 0.81 for the affection component, and 0.89 for behavioral tendency.

### Research Object and Sampling Data

With experimental research, 386 high school students in Taiwan completed the 16-week (3 h per week for a total of 48 h) flipped learning. The data collected through questionnaires were analyzed with SPSS and further analysis of variance and regression analysis were utilized for testing the formulated hypotheses.

### Statistical Tools Used

In this study, analysis of variance was applied to discuss the difference of flipped learning in learning motivation and learning attitude, and regression analysis was used for understanding the relationship between learning motivation and learning attitude of high school students.

## Analysis of Data and Testification of Hypotheses

### Effects of Flipped Learning on Learning Motivation and Learning Attitude

#### Differential Analysis of Flipped Learning in Learning Motivation

We can see from Table 1 that the difference of flipped learning in learning motivation reveals a significant difference of flipped learning in intrinsic orientation, where flipped learning (4.33) shows a higher level of intrinsic orientation than traditional teaching (3.64) and similarly, flipped learning (4.18) shows a higher level of extrinsic orientation than traditional teaching (3.77). Hence, H1 is supported.

**TABLE 1:** Differential analysis of flipped learning in learning motivation.

**Variable**		***F***	***p***	**Scheffe *post hoc***
Flipped learning	Intrinsic orientation	18.627	0.000*	Flipped learning (4.33) > traditional teaching (3.64)
	Extrinsic orientation	25.439	0.000*	Flipped learning (4.18) > traditional teaching (3.77)

The symbol * stands for *p* < 0.05.

#### Difference Analysis of Flipped Learning in Learning Attitude

It is inferred from Table 2 that the difference of flipped learning in learning attitude reveals a remarkable difference of flipped learning in the cognition component, where flipped learning (4.24) shows a higher level of the cognition component than traditional teaching (3.58), likewise flipped learning (4.05) shows a higher level of the affection component than traditional teaching (3.35), and similarly, flipped learning (4.46) shows a higher level of behavioral tendency than traditional teaching (3.98). Hence, H2 is supported.

**TABLE 2:** Differential analysis of flipped learning in learning attitude.

**Variable**		***F***	***p***	**Scheffe *post hoc***
Flipped learning	Cognition component	21.577	0.000*	Flipped learning (4.24) > traditional teaching (3.58)
	Affection component	27.962	0.000*	Flipped learning (4.05) > traditional teaching (3.35)
	Behavioral tendency	33.125	0.000*	Flipped learning (4.46) > traditional teaching (3.98)

The symbol * stands for *p* < 0.05.

### Relationship Between Learning Motivation and Learning Attitude

#### Relationship Between Learning Motivation and Cognition Component

It is learnt from Table 3 that the results of regression analysis reveal a notable effect of intrinsic orientation (β = 2.287^∗∗^) and extrinsic orientation (β = 2.436^∗∗^) on the cognition component of learning attitude. Hence, H3 is supported.

**TABLE 3:** Relationship between learning motivation and learning attitude.

**Learning motivation (independent variable)**	**Learning attitude (dependent variable**)					
	**Cognition component**		**Affection component**		**Behavioral tendency**	
	**β**	***p***	**β**	***p***	**β**	***p***
Intrinsic orientation	2.287**	0.000	2.155**	0.000	2.382**	0.000
Extrinsic orientation	2.436**	0.000	2.217**	0.000	2.537**	0.000
*F*	27.162		34.859		37.281	
Significance	0.000***		0.000***		0.000***	
*R* ^2^	0.263		0.335		0.361	
Adjusted *R*^2^	0.248		0.314		0.342	

The symbols ** stands for *p* < 0.01 and *** stands for *p* < 0.001. Self-organized for this study.

#### Relationship Between Learning Motivation and Affection Component

It is understood from Table 3 that the results of regression analysis reveal significant effects of intrinsic orientation (β = 2.155^∗∗^) and extrinsic orientation (β = 2.217^∗∗^) on the affection component of learning attitude. Hence, H4 is supported.

#### Relationship Between Learning Motivation and Behavioral Tendency

It is inferred from Table 3 that the results of regression analysis reveal remarkable effects of intrinsic orientation (β = 2.382^∗∗^) and extrinsic orientation (β = 2.537^∗∗^) on behavioral tendency of learning attitude. Hence H5 is supported.

## Results of the Study

The results of the study reveal that the students who were taught using “flipped teaching models” demonstrate better levels of comprehension due to changes in learning style and attitude in the flipped classroom, which enhances their effectiveness in learning language than their counter arts who received traditional teaching.

The results of the differential analyses with regard to the dimensions of “learning motivation” reveal that there is a remarkable difference indicating that the flipped learning group possessed higher levels of intrinsic orientation (4.33) and extrinsic orientation (4.18) than the traditional learning group (3.64 and 3.77), respectively, in their “learning motivation.”

The results of the differential analyses with regard to the dimensions of “learning attitude” reveal that there is a remarkable difference indicating that the flipped learning group possessed higher levels of the cognition component (4.24), affection component (4.05), and behavior tendency (4.46) than the traditional learning group (3.58, 3.35, and 3.98), respectively, in their “learning attitude”.

The results of the regression analyses with regard to the effects of “learning motivation” on “learning attitude” are that (i) there are remarkable positive effects of intrinsic orientation (β = 2.287) and extrinsic orientation (β = 2.436) on the cognition component of learning attitude, (ii) there are remarkable positive effects of intrinsic orientation (β = 2.155) and extrinsic orientation (β = 2.217) on the affection component of learning attitude, and (iii) there are remarkable positive effects of intrinsic orientation (β = 2.382) and extrinsic orientation (β = 2.537) on behavioral tendency of learning attitude. While comparing the “adjusted *R*^2^ values,” it is found that the effects of intrinsic orientation and extrinsic orientation of learning motivation on behavioral tendency (0.342) are greater than that of the cognitive component (0.248) and affection component (0.314) of “learning attitude”.

## Discussion

Students’ low performance on language occurs slowly over time. In this case, a series of basic teaching initiatives are required to enhance low-performance students’ language learning effectiveness step by step. Instructors could allocate suitable films with new language learned in the unit according to students’ individual ability to help low-performance learners’ language learning effectiveness. Students from different levels generating discussions in the same group could easily result in “hitchhike.” In this case, group competition could be used in real teaching for students who care about their performance to be able to balance the group performance. In this case, students with better performance are willing to help low-performance students attain better learning motivation and learning attitude.

## Conclusion

The research results show notable differences in language learning between the experimental group and the control group after the experimental teaching. Students in the experimental group present significantly higher language learning motivation and attitude than those in the control group. It reveals that flipped learning could help low-performance students enhance language learning effectiveness. In other words, flipped learning, compared to traditional teaching, could enhance students’ learning motivation and learning attitude and because of these reasons flipped learning is certainly worth attempting. However, traditional teaching also maintains some advantages for it to remain and be alternatively used with flipped learning. With flipped learning, students feel that the teaching content is easier to learn and internalize. Furthermore, flipped learning allows students to discuss topics with each other and teachers to guide their learning.

## Data Availability Statement

The original contributions presented in the study are included in the article/supplementary material, further inquiries can be directed to the corresponding author/s.

## Ethics Statement

The present study was conducted in accordance with the recommendations of the Ethics Committee of Chung Yuan Christian University, with written informed consent being obtained from all the participants. All the participants were asked to read and approved the ethical consent form before participating in the present study. The participants were also asked to follow the guidelines in the form in the research. The research protocol was approved by the Ethical Committee of Chung Yuan Christian University.

## Author Contributions

C-PC performed the initial analyses and wrote the manuscript. K-WC and C-JH assisted in the data collection and data analysis. All authors revised and approved the submitted version of the manuscript.

## Conflict of Interest

The authors declare that the research was conducted in the absence of any commercial or financial relationships that could be construed as a potential conflict of interest.

## Publisher’s Note

All claims expressed in this article are solely those of the authors and do not necessarily represent those of their affiliated organizations, or those of the publisher, the editors and the reviewers. Any product that may be evaluated in this article, or claim that may be made by its manufacturer, is not guaranteed or endorsed by the publisher.

## References

[B1] Alexander M. M. (2018). The flipped classroom: engaging the student in active learning. *J. Legal. Stud. Educ.* 35 277–300. 10.1111/jlse.12078

[B2] Awidi I. T. Paynter M. (2019). The impact of a flipped classroom approach on student learning experience. *Comput. Educ.* 128 269–283. 10.1016/j.compedu.2018.09.013

[B3] Bakla A. (2018). Learner-generated materials in a flipped pronunciation class: a sequential explanatory mixed-methods study. *Comput. Educ.* 125 14–38. 10.1016/j.compedu.2018.05.017

[B4] Chang C. Y. Sung H. Y. Guo J. L. Chang B. Y. Kuo F. R. (2019). Effects of spherical video-based virtual reality on nursing students’ learning performance in childbirth education training *Interactive Learn. Environ* 27, 1–17. 10.1080/10494820.2019.1661854

[B5] Chang S. C. Hwang G. J. (2018). Impact of an augmented reality-based flipped learning guiding approach on students’ scientific project performance and perceptions. *Comput. Educ.* 125 226–239. 10.1016/j.compedu.2018.06.007

[B6] Chen M. R. A. Hwang G. J. Chang Y. Y. (2019). A reflective thinking−promoting approach to enhancing graduate students’ flipped learning engagement, participation behaviors, reflective thinking and project learning outcomes. *Br. J. Educ. Technol.* 50 2288–2307. 10.1111/bjet.12823

[B7] Cheng K. H. Tsai C. C. (2019). A case study of immersive virtual field trips in an elementary classroom: Students’ learning experience and teacher-student interaction behaviors. *Comput. Educ.* 140:103600.

[B8] Chukwuemeka E. J. Dominic S. Kareem M. A. Mailafia I. A. (2021). Redesigning educational delivery systems: the needs and options for continuous learning during the coronavirus (COVID-19) pandemic in nigeria. *Contemp. Educ. Technol.* 13:e292. 10.30935/cedtech/9363

[B9] Drozdikova-Zaripova A. R. Sabirova E. G. (2020). Usage of digital educational resources in teaching students with application of “Flipped classroom” technology. *Contemp. Educ. Technol.* 12:e278. 10.30935/cedtech/8582

[B10] Green L. S. (2019). Flipped learning environments: an introduction for librarians who design and teach. *Lib. Technol. Rep.* 55 11–16.

[B11] Karabulut I. A. Jaramillo Cherrez N. Jahren C. T. (2018). A systematic review of research on the flipped learning method in engineering education. *Br. J. Educ. Technol.* 49 398–411. 10.1111/bjet.12548

[B12] Lin C. J. Hwang G. J. Fu Q. K. Chen J. F. (2018). A flipped contextual game-based learning approach to enhancing EFL student’s English business writing performance and reflective behaviors. *J. Educ. Technol. Soc.* 21 117–131.

[B13] Nerantzi C. (2020). The use of peer instruction and flipped learning to support flexible blended learning during and after the COVID-19 Pandemic. *Int. J. Manage. Appl. Res.* 7 184–195.

[B14] Umutlu D. Akpinar Y. (2020). Effects of different video modalities on writing achievement in flipped english classes. *Contemp. Educ. Technol.* 12:e270. 10.30935/cedtech/7993

[B15] Zhang S. L. (2019). Chinese-as-a-foreign-language learners’ use of self-regulated learning in flipped/blended learning environments - a descriptive study. *Stud. Self Access Learn. J.* 10 181–204. 10.37237/100205

